# Prey diversity as a driver of resource partitioning between river‐dwelling fish species

**DOI:** 10.1002/ece3.2793

**Published:** 2017-02-26

**Authors:** Javier Sánchez‐Hernández, Heidi‐Marie Gabler, Per‐Arne Amundsen

**Affiliations:** ^1^ Department of Arctic and Marine BiologyFaculty of BiosciencesFisheries and EconomicsUiT The Arctic University of NorwayTromsøNorway; ^2^ Department of Zoology, Genetics and Physical AnthropologyFaculty of BiologyUniversity of Santiago de CompostelaSantiago de CompostelaSpain

**Keywords:** biodiversity, coexistence, dietary overlap, interindividual variation, mixed models, niche theory

## Abstract

Although food resource partitioning among sympatric species has often been explored in riverine systems, the potential influence of prey diversity on resource partitioning is little known. Using empirical data, we modeled food resource partitioning (assessed as dietary overlap) of coexisting juvenile Atlantic salmon (*Salmo salar*) and alpine bullhead (*Cottus poecilopus*). Explanatory variables incorporated into the model were fish abundance, benthic prey diversity and abundance, and several dietary metrics to give a total of seventeen potential explanatory variables. First, a forward stepwise procedure based on the Akaike information criterion was used to select explanatory variables with significant effects on food resource partitioning. Then, linear mixed‐effect models were constructed using the selected explanatory variables and with sampling site as a random factor. Food resource partitioning between salmon and bullhead increased significantly with increasing prey diversity, and the variation in food resource partitioning was best described by the model that included prey diversity as the only explanatory variable. This study provides empirical support for the notion that prey diversity is a key driver of resource partitioning among competing species.

## 1 Introduction

Resource partitioning, assumed to be a principal mediator of biodiversity, has been central for understanding how a community of species persists over time. Consumer interactions have generally been viewed from the perspective of predator diversity (e.g., Griffin, Haye, Hawkins, Thompson, & Jenkins, 2008; Ives, Cardinale, & Snyder, 2005; Northfield, Snyder, Ives, & Snyder, 2010), with the diversity of prey species rarely being taken into account (but see Duffy et al., 2007).

Biodiversity of the prey community could be important in food resource partitioning, because increased prey diversity should enhance the possibility of interactive segregation in resource utilization (Hillebrand & Matthiessen, 2009; Hillebrand & Shurin, 2005). Thus, it is pertinent to ask whether there is a decrease in competition for food among species when prey diversity is high. That is, does prey diversity influence competitive interactions and food resource partitioning between sympatric species? If prey diversity is high, sympatric species may be able to segregate in resource use and partitioning may occur, as predicted by niche theory (Schoener, 1974, 1989). Alternatively, if prey diversity is low, sympatric species may utilize the same resources, and niche overlap will be high or competitive exclusion could occur (Keddy, 2001; Schoener, 1989). The potentially important relationship between prey diversity and dietary overlap between sympatric species has rarely been explored, but the few examples that exist from fish (Barili, Agostinho, Gomes, & Latin, 2011; Targett, 1981; Wuellner et al., 2011) and other vertebrates (Jiang, Feng, Sun, & Wang, 2008; Martin & Garnett, 2013; Zapata, Travaini, Delibes, & Martinez‐Peck, 2005) indicate that increased prey diversity may mitigate competition through enhanced resource partitioning (but see Wuellner et al., 2011). It should, however, be kept in mind that variables other than prey diversity, such as prey abundance, foraging mode, diel patterns, and habitat segregation for feeding, may also be major determinants of food resource partitioning (e.g., Crow, Closs, Waters, Booker, & Wallis, 2010; Kronfeld‐Schor & Dayan, 2003; Nakano, Fausch, & Kitano, 1999; Sánchez‐Hernández, Vieira‐Lanero, Servia, & Cobo, 2011). Consequently, the study of food resource partitioning requires a framework that includes the complex interplay among prey diversity, prey abundance, fish abundance, and diet variation.

We examined the relationship between several possible explanatory variables (prey diversity, prey abundance, fish abundance, and diet variation of species) and food resource partitioning (measured as dietary overlap) of coexisting juvenile Atlantic salmon (*Salmo salar* Linnaeus, 1758; henceforth salmon) and alpine bullhead (*Cottus poecilopus* Heckel, 1836; henceforth bullhead). We used these two fishes as model species because their feeding ecology and competitive interactions are well documented (Amundsen & Gabler, 2008; Gabler & Amundsen, 1999, 2010). Both species feed on similar prey with a preference for benthic invertebrates (Gabler & Amundsen, 1999), and they are presumed to be resource competitors because their diets and habitat use are similar even when food resources are limited (Amundsen & Gabler, 2008; Gabler & Amundsen, 1999, 2010). Further, the two species do not show significant diel segregation in feeding in subarctic rivers (Gabler & Amundsen, 1999). This provides an opportunity to examine prey choice and food resource partitioning in sympatric fish species by comparing multiple sites that differ in prey diversity, prey abundance, and fish density. The main objectives were to explore (i) whether food resource partitioning occurred between the two species and (ii) whether prey diversity or any other of the potential explanatory variables could be identified as significant predictors of food resource partitioning. We hypothesized that food resource partitioning would increase with increasing prey diversity irrespective of other site‐specific characters.

## 2 Materials and methods

### 2.1 Study area

**Figure 1:**
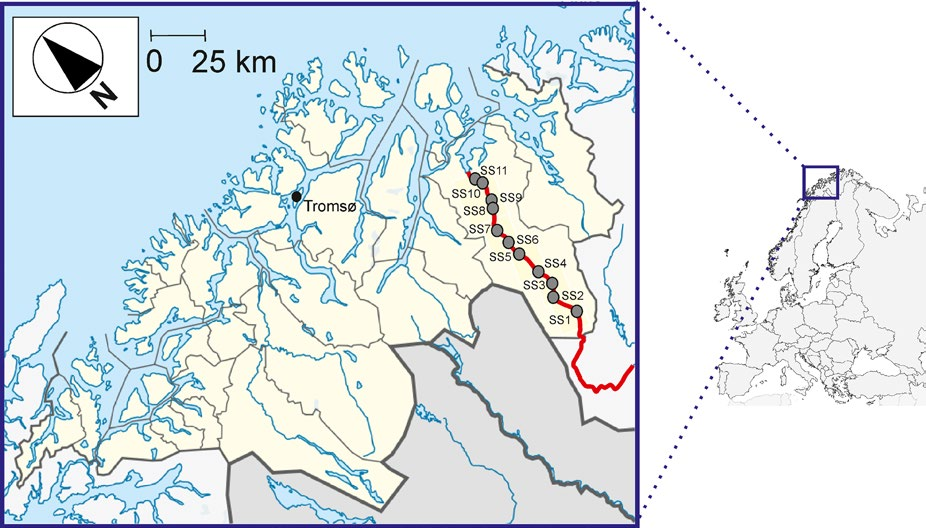
Location of the River Reisa (in red), northern Norway, showing the sampling sites (SS, gray circle) labeled from the upper part (SS1) to lower part (SS11)

The study was carried out in River Reisa (Figure 1), a subarctic, oligotrophic river in northern Norway (latitude 69°N). The river, approximately 140 km long and around 40 m wide along the studied sections, drains a catchment area of 2,516 km². The river does not have any significant flow regulation structure, and the mean annual discharge is 34 m³/s with the water flow typically peaking at 200–250 m³/s in late June (Gabler & Amundsen, 1999). The Reisa National Park is located in the headwater of the Reisa basin, and the park and surrounding areas provide grazing for semidomesticated reindeer. The Reisa basin includes a mixture of grass paddocks and forest [birch (*Betula pubescens* Ehrh.) and scattered pine (*Pinus sylvestris* L.)], with small rural areas interspersed in the lower part. Thus, agriculture, stockbreeding, and domestic sewage effluents are the primary but modest human impacts on the catchment. The climate is typically subarctic with long, dark, and cold winters, and the river is usually ice‐covered from November until April. Geologically, the study basin is characterized by an accumulation of granite and gneiss, and boulders, cobble, and gravel constitute the main substrates of the river bottom. The riparian vegetation is chiefly composed by deciduous woodland (birch) and pine forests. No information is available about drift patterns or magnitude of terrestrial subsidies into the River Reisa. It should be noted, however, that the contribution of terrestrial insects to the drift in Norwegian subarctic rivers may be very noticeable from June to October (Johansen, Elliott, & Klemetsen, 2000). In fact, terrestrial insects are the largest group in the drift of another northern Norwegian river (River Saeterelva, latitude 68°N) in August, but with very low densities in May (Johansen et al., 2000).

River Reisa supports recreational salmon angling and the annual reported catches of salmon over the last 20 years have varied greatly from a few hundred kg to nearly 12,000 kg. Catches were particularly high from 2008 to 2011 (approximately 8,000–12,000 kg/year) (Svenning, 2011). The distribution of the bullhead coincides with that of salmon, and these fish are the dominant species in the fish community of the river. Other fish species, such as Arctic charr *Salvelinus alpinus* (Linnaeus, 1758), brown trout *Salmo trutta* Linnaeus, 1758, and three‐spine stickleback *Gasterosteus aculeatus* Linnaeus 1758, are also present in the river basin (Gabler & Amundsen, 2010). A natural waterfall located about 90 km from the sea represents the upstream limit to migrating fish.

### 2.2 Sampling

Sampling protocols used in this study conform to the ethical laws of the country. Based on previous knowledge of the study area (e.g., Amundsen & Gabler, 2008; Gabler & Amundsen, 1999, 2010), the sampling design was performed to match with the distribution of the model species as well as to ensure variations in biotic conditions (fish and benthic invertebrates) among sampling sites along the River Reisa. Sampling of fish and benthic invertebrates was conducted at 11 sites along the main course of the river in August 2004 (Figure 1). August is the time when the aquatic food resource supply is lowest relative to the energetic requirements of salmon and bullheads and thus the period when competitive interactions should be strongest (Amundsen, Bergersen, Huru, & Heggberget, 1999; Amundsen & Gabler, 2008; Amundsen, Gabler, Herfindal, & Riise, 2000; Gabler & Amundsen, 2010). Some of the study sections were relatively close to each other; the minimum distance apart was between SS11 and SS10 and was approximately 3 km, whereas the maximum distance apart was about 10 km between SS5 and SS4 (Figure 1). We assumed fish movement between sampling sites would be negligible, and the study sections were deemed independent. Data from a published study (Gabler & Amundsen, 1999) with monthly sampling during the ice‐free season were included in the analyses to examine for possible seasonal variations in the prey diversity food resource partitioning relationship. These data were collected following the same sampling protocol as in the present study, allowing direct comparison between studies.

Fish and benthic invertebrate samples were collected from riffles with cobble and gravel as the main substrate. Prior to sampling, site selection was visually performed to ensure habitat similarity among sampling sites to diminish any possible error in the results related to field sampling techniques such as bias in fish removal rate among sampling sites. Thus, habitat conditions among sampling sites were deemed similar, but no specific habitat measurements were taken.

Benthic invertebrates were collected at each site to study the prey availability. Samples were collected immediately after fish sampling near to where electrofishing was conducted. Three parallel samples were taken using the kicking method (Williams & Feltmate, 1992), standardized by kicking for 3 min inside a metal frame defining 1.5 × 1.5 m of the bottom. Benthic invertebrates were sorted and identified to the lowest taxon possible, and prey abundance was calculated as number of individuals per m^2^. Plecoptera and Ephemeroptera nymphs and Trichoptera larvae were identified to species level, and other taxa to the genus or family level. Prey diversity (*H*ʹ) was calculated as Shannon's diversity index (Shannon & Weaver, 1949):

(1) $$H^{\prime }=-\sum p_{i}\log_{10}p_{i}$$

where *p*_*i*_ is the proportion of species *i* in the benthic invertebrate samples.

Fish were collected using portable backpack electrofishing gear with pulsed direct current (GeOmega backpack model; 700–1,400 V, 5 A maximum intensity, 40–80 Hz) and a single anode of 30 cm diameter. Three‐pass removal electrofishing was conducted at each sampling site with 30 min between passes following the standardized procedures described for the EU Water Framework Directive (European Commission, 2000) by the CEN directive on fishing with electricity in wadeable rivers (CEN, 2003). However, due to large river widths and depths, no nets were used to block the upstream and downstream boundaries. Fish sampling was conducted in an upstream direction from the riverbank to a water depth of about 70 cm over a stream section of 100 m. Each fish was identified, measured (fork length, mm), and preserved in 96% ethanol for later dissection and dietary analysis. Although the depletion method with only three passes may be inadequate to estimate fish abundance, particularly in sampling events with low capture probabilities (e.g., Dorazio, Jelks, & Jordan, 2005), the abundance of each fish species (here overall fish density regardless of fish length) at each site was estimated as number of fish per 100 m^2^ using Zippin multiple‐pass depletion method (Zippin, 1956). Although this fish density estimation might be rough, it is assumed to provide representative estimates of the relative fish abundance among sampling sites. Fish abundances are given in Table 1.

**Table 1:** Food resource partitioning (measured as dietary overlap, %) between Atlantic salmon parr and alpine bullhead, prey availability (prey diversity— measured as Shannon's diversity index, and abundance—estimated as ind./m^2^), fish abundance (fish/100 m^2^), and dietary metrics of the two fish species (Levins’ index, individual dietary specialization, and surface prey contribution) from the different sampling sites (SS) in the Reisa River. 1‐*IS* = prevalence of individual dietary specialization, where 1‐*IS* is given as mean ± *SD*. Alpine bullhead (bul), Atlantic salmon (sal), contribution of surface prey in the diet (surface). Overlap total = dietary overlap calculated using all prey. Overlap aquatic = dietary overlap calculated without surface prey

	Sampling sites										
	SS1	SS2	SS3	SS4	SS5	SS6	SS7	SS8	SS9	SS10	SS11
Prey resources											
Diversity	0.74	0.94	0.78	0.50	0.70	0.98	0.67	0.75	0.77	0.79	0.90
Abundance	393.2	304	82.2	95.3	63.6	83.3	120.1	123.1	63.6	144.6	77.9
Fish abundance											
Alpine bullhead	36.6	35.1	1.9	20	28.6	22.6	0.9	19.4	2.8	18.4	2
Atlantic salmon	5.3	2.5	8.9	2.9	0.001	0.001	16.3	3.9	8.9	4.4	3.9
Brown trout	0	0	1.14	0.70	0	0	5.70	4.96	4.61	1.90	3.82
Arctic charr	0	1.53	0	0	0	0	0.79	5.86	0.82	4.43	1.27
Total	41.90	39.13	11.94	23.60	28.60	22.60	23.73	34.10	17.14	29.13	10.99
Diet											
Levins (bul)	3.0	5.3	5.9	4.2	4.8	6.9	3.9	5.8	6.8	5.3	6.2
Levins (sal)	5.8	2.2	9.0	5.6	4.1	6.6	4.3	5.4	2.8	1.9	3.6
1‐*IS* (bul)	0.54 ± 0.17	0.64 ± 0.18	0.59 ± 0.11	0.62 ± 0.17	0.61 ± 0.16	0.80 ± 0.10	0.49 ± 0.16	0.69 ± 0.14	0.76 ± 0.12	0.66 ± 0.13	0.64 ± 0.13
1‐*IS* (sal)	0.47 ± 0.04	0.42 ± 0.22	0.50 ± 0.08	0.64 ± 0.23	0.54 ± 0.13	0.75 ± 0.09	0.58 ± 0.17	0.61 ± 0.13	0.46 ± 0.16	0.45 ± 0.21	0.63 ± 0.06
Surface (bul)	0	0	0	4.6	9.5	1.8	0	0	0.9	0.2	0
Surface (sal)	0	65	11.7	6	42.5	0	0	0	0	20	30
Overlap total	54.9	11.5	31.7	62.5	31.6	33.5	34.4	44.6	33.2	41.3	25.3
Overlap aquatic	54.9	44.0	37.5	65.4	57.4	34.4	35.5	44.6	33.6	51.4	40.3
Sampling size											
Alpine bullhead (*n*)	37	69	5	57	32	32	9	43	17	29	11
Atlantic salmon (*n*)	9	8	16	12	13	9	61	20	10	7	14

To avoid bias resulting from possible differences in feeding behavior of different size classes of fish (e.g., Dineen, Harrison, & Giller, 2007; Hesthagen, Saksgård, Hegge, Dervo, & Skurdal, 2004), only individuals <100 mm were used for diet analysis. In total, 179 salmon parr and 341 bullheads were caught, of which 142 salmon and 341 bullheads were used for stomach content analyses (SCA). We attempted to collect at least ten individuals of each fish species from each sampling site. Although this goal was not always achieved for salmon (successful in seven of eleven sampling sites) and bullhead (nine of eleven sampling sites; see sampling sizes of each locality in Table 1), we assume that the captured individuals are representative of the entire population. Additionally, to dismiss any possible impact of the unequal sampling sizes, we generated 1,000 bootstrap samples (see Section 2.3 below).

**Figure 2:**
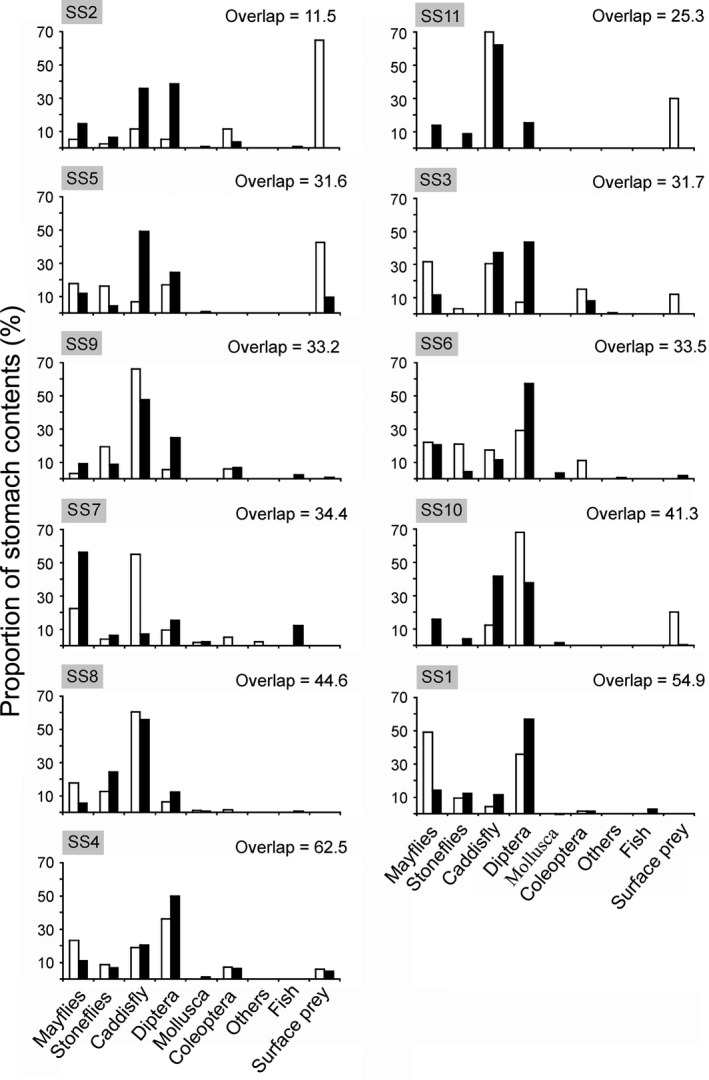
Proportion of different prey groups in the stomach contents of Atlantic salmon parr (white bars) and alpine bullhead (black bars) (the category “others” includes chydorids, water mites, and unidentified prey taxa). Data are presented for each sampling site ranked from the highest to the lowest food resource partitioning (dietary overlap value). The presented dietary overlap values are calculated with all prey types included (i.e., with the highest taxonomical resolution as in Table S2)

The stomachs were opened, and the percentage of total fullness was visually determined, ranging from empty (0%) to full (100%) (see subjective methods in Hyslop, 1980). Each prey item was then identified to the same taxonomic level as for the benthic invertebrate samples. The relative contribution of each prey to the total stomach fullness was estimated according to Amundsen, Gabler, and Staldvik (1996). That is, the sum of all prey categories of a stomach meets the visually determined total fullness. In mathematical terms, the contribution of each prey to the diet is described as percent prey abundance (*A*_*i*_):

(2) $$A_{i}=100\sum_{i=1}^{n}S_{i}{\left(\sum_{i=1}^{n}S_{t}\right)}^{-1}$$

where *S*_*i*_ is stomach fullness of prey type *i*,* S*_*t*_ is the total fullness of all prey categories, and *n* is the number of fish with prey *i* in the stomach. For the graphical representation, prey typically caught at the water surface including biting midges (*Culicoides* spp.), aerial stages of aquatic insects, spiders, and unidentified terrestrial insects were combined and designated as “surface prey.” Similarly, the aquatic taxa were grouped into seven prey categories (Ephemeroptera, Plecoptera, Trichoptera, Diptera, Mollusca, Coleoptera, and others) for the plotting of the diet graphs (Figure 2).

Dietary overlap (*P*_*jk*_) was calculated as percentage overlap (Krebs, 1989) using the lowest taxonomic resolutions of prey:

(3) $$P_{jk}=\left[\sum_{1}^{n}\left(\text{minimum}\;A_{ij},\;A_{ik}\right)\right]$$

where *P*_*jk*_ is the percentage overlap between species *j* and *k*, and *A*_*ij*_ and *A*_*ik*_ are the percent prey abundance of resource *i* used by species *j* and *k*,* n* is the total number of resource categories. Prey diversity analyses were restricted to benthic invertebrates, so dietary overlaps were also calculated and analyzed without inclusion of surface prey.

We addressed the trophic niche at the population level by estimating niche breadth (*B*) using Levins’ index (Levins, 1968):

(4) $$B=1/\sum P_{i}^{2}$$

where *P*_*i*_ is the proportion of each prey type *i* in the diet expressed as fraction rather than percentage (Amundsen, Knudsen, & Bryhni, 2010).

To study individual dietary specialization, the proportional similarity (*PS*_*i*_) index was calculated (Bolnick, Yang, Fordyce, Davis, & Svanbäck, 2002):

(5) $$PS_{i}=1-0.5\left|P_{ij}-Q_{j}\right|=\sum \left(P_{ij},Q_{j}\right)$$

where *P*_*ij*_ is the proportion of resource category *j* in the diet of individual *i*, and *Q*_*j*_ the proportion of resource category *j* in the diet of the population. This index compares each individual's diet to that of the population, with values ranging between 0 and 1. For individuals that specialize on a single or few prey types, *PS*_*i*_ values are low, whereas for individuals that consume resources in a similar proportion to the population as a whole, *PS*_*i*_ values approach 1 (Bolnick et al., 2002). The overall prevalence of individual specialization was calculated as the inverse of the average individual *PS*_*i*_ values (Quevedo, Svanbäck, & Eklöv, 2009).

### 2.3 Statistical analyses

The relationships between dietary overlap (henceforth food resource partitioning) and the biotic variables (explanatory variables) were investigated with linear mixed‐effect models using sampling site as a random factor. The data were hierarchically structured with explanatory variables being nested within sampling sites, and mixed‐effect models were used to account for potential random effects among sampling sites. Thus, the random part contains components that allow for heterogeneity of variables among the studied sampling sites. Seventeen potential explanatory variables of food resource partitioning were considered (Table 2). First, we selected fixed terms (i.e., explanatory variables that are deterministic) that describe the response variable *Y* (here food resource partitioning) as a function of the explanatory variables. The optimal fixed component was established based on a stepwise forward selection method (*step* function). This procedure enabled us to select which explanatory variables are significant, and which are not. This selection was made according to Akaike information criteria (AIC) (Akaike, 1974). Nine explanatory variables were selected for model simulations (see significant explanatory variables in Table 2). Next, we built models based on the nine selected explanatory variables using the restricted maximum likelihood (REML) estimation for linear regression models. REML aims to correct the estimator for the variance, and as suggested by Zuur, Ieno, Walker, Saveliev, and Smith (2009), this procedure should be used to fit models with many fixed terms (here *n *=* *9). Model selection was also established using AIC. When sample size is small or the number of parameters is large, AIC_c_ (AIC corrected for small‐sample bias) or QAIC_c_ (AICc for overdispersed data) should be used instead of AIC (Anderson & Burnham, 2002). In the present study AIC_c_ was used for model selection, with the best model being the one with the lowest AIC_c_ values. The strength of association between food resource partitioning and explanatory variables from the best models was tested using Pearson's rank correlation. Finally, we ran sensitivity analyses to test whether linear mixed‐effect models were the same after excluding surface prey from the dietary analyses. A significance level of *p *=* *.05 was used in all analyses. Models were performed using R 3.2.2 (R Core Team 2015) using “nlme” (Pinheiro, Bates, DebRoy, & Sarkar, 2016) and “MuMIn” (Bartoń, 2016) packages. The bootstrapping technique was performed using the “ boot” package (Canty & Ripley, 2016) employing techniques outlined in Zuur et al. (2009) for an additional test of the model. We applied a parametric bootstrap (*n *=* *1,000) on the best linear mixed‐effects model explaining variation of food resource partitioning between Atlantic salmon parr and alpine bullhead. The model was applied on the bootstrapped data following the same modeling procedures as described above. Residuals of the final selected model (original data, bootstrapped data, and sensitivity analyses) were visually inspected for deviations from normality and heteroscedasticity, without finding any evidence for violation of model assumptions (see Fig. S1).

**Table 2:** Full list of explanatory variables used to explore their possible influence of on food resource partitioning (measured as dietary overlap) between juvenile Atlantic salmon (*Salmo salar*) and alpine bullhead (*Cottus poecilopus*). Significant explanatory variables after stepwise variable selection (*). Pearson's rank correlation between each explanatory variable and food resource partitioning is shown (significant ones marked in bold)

Explanatory variables	Definition	Correlation
Prey diversity*	Macrozoobenthos diversity calculated as Shannon's diversity index	R = −.73, ***p *** **=** *** *** **.011**
Prey abundance*	Macrozoobenthos abundance estimated as ind./m^2^	R = .07, *p *=* *.831
Atlantic salmon abundance*	Density (fish/100 m^2^) of Atlantic salmon parr	R = −.01, *p *=* *.992
Alpine bullhead abundance*	Density (fish/100 m^2^) of alpine bullhead	R = .14, *p *=* *.684
Brown trout abundance	Density (fish/100 m^2^) of brown trout	R = −.04, *p *=* *.895
Arctic charr abundance*	Density (fish/100 m^2^) of Arctic charr	R = .03, *p *=* *.934
Total fish abundance	Total fish community density (fish/100 m^2^)	R = .18, *p *=* *.601
Surface prey (Atlantic salmon)*	Contribution of surface prey in the diet of Atlantic salmon parr	R = −.67, ***p *** **=** *** *** **.024**
Surface prey (alpine bullhead)	Contribution of surface prey in the diet of alpine bullhead	R = .15, *p *=* *.666
Niche breadth (Atlantic salmon)	Levins’ index of Atlantic salmon parr	R = .32, *p *=* *.330
Niche breadth (alpine bullhead)*	Levins’ index of alpine bullhead	R = −.50, *p *=* *.120
Individual specialization (Atlantic salmon)	Individual dietary specialization of Atlantic salmon parr	R = .22, *p *=* *.508
Individual specialization (alpine bullhead)	Individual dietary specialization of alpine bullhead	R = −.16, *p *=* *.628
Stomach fullness (Atlantic salmon)	Stomach fullness (%) of Atlantic salmon parr	R = .54, *p *=* *.085
Stomach fullness (alpine bullhead)	Stomach fullness (%) of alpine bullhead	R = .08, *p *=* *.818
Size (Atlantic salmon)*	Fork length (mm) of Atlantic salmon parr	R = −.50, *p *=* *.114
Size (alpine bullhead)*	Fork length (mm) of alpine bullhead	R = −.24, *p *=* *.484

## 3 Results

### 3.1 Prey resources

Prey diversity varied widely among localities, with the Shannon index ranging from 0.50 to 0.98, and prey abundances varied among sampling sites, ranging from 63.1 to 393.2 ind./m^2^ (Table 1). Chironomidae was usually the most abundant taxon, but in some localities *Baetis* spp., *Ephemerella aurivillii* (Bengtsson), and *Capnia* sp. were numerically dominant (taxa recorded in benthic invertebrate samples are given in Table S1).

### 3.2 Food resource partitioning

Both salmon and bullhead fed mainly on benthic invertebrates (Figure 2), but differences were found between the species and among localities in the contributions of the different prey taxa to the diet. In general, Ephemeroptera nymphs and larval Diptera and Trichoptera dominated the diet of both fish species, with abundance values ranging between 21.3% and 94.7%. Surface prey was an important dietary component for salmon in some localities (Figure 2) (details of stomach content analyses are given in Table S2).

**Figure 3:**
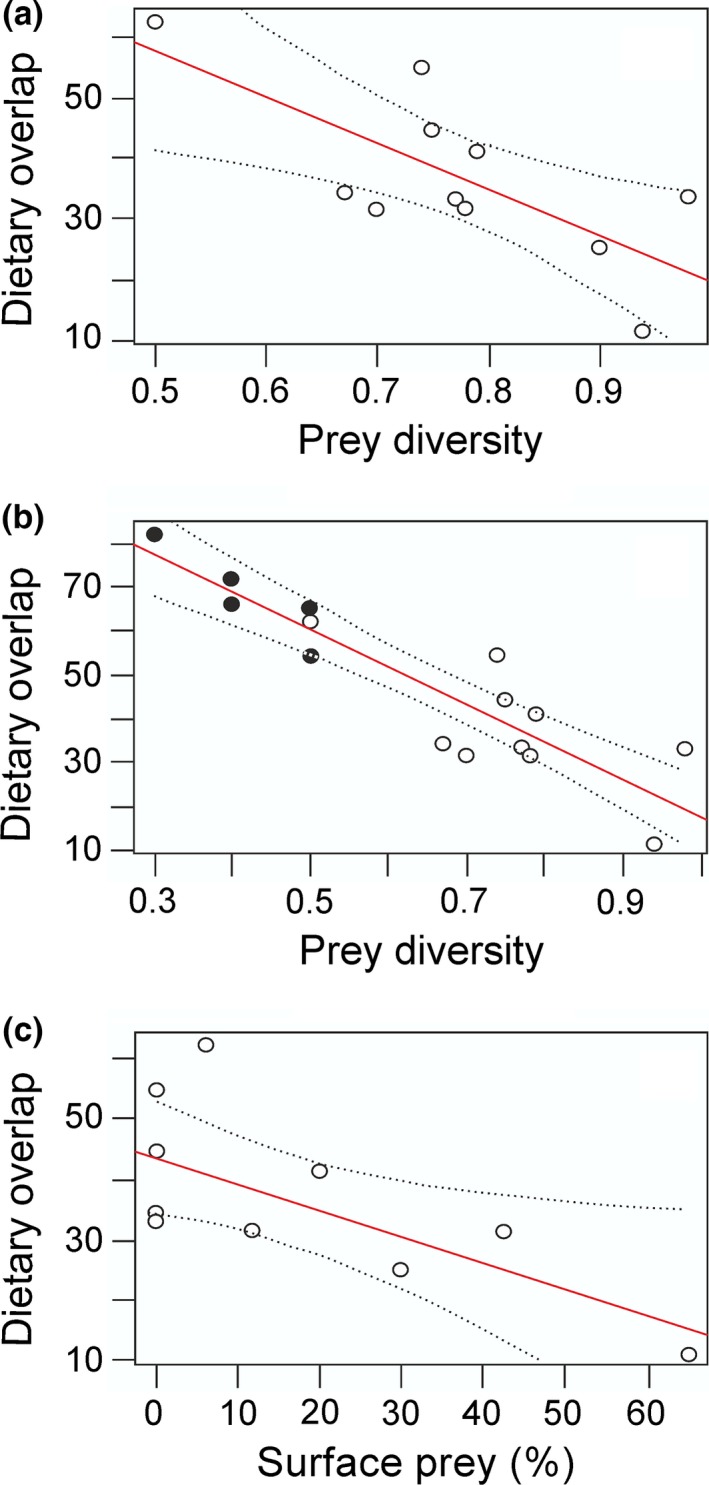
Relationship between prey diversity and food resource partitioning (measured as dietary overlap) between Atlantic salmon parr and alpine bullhead at (a) eleven sites in River Reisa, (b) with data on seasonal variation included (filled circles), (c) between abundance of surface prey in the diet of Atlantic salmon parr and food resource partitioning. Both food resource partitioning and prey diversity have been estimated with the highest taxonomical resolution of the prey. Significant linear trends with 95% confidence limits are shown

Mean dietary overlap between salmon and bullhead was 36.8%, but overlap varied quite widely among sampling sites, ranging from 11.5% to 62.5% (Table 1). A model that included prey diversity as the only explanatory variable was the best one, having the lowest AIC_c_ value (Table S3), and parameters of this model are given in Table 3. Dietary overlap exhibited a significant negative correlation with prey diversity (Figure 3a; R = −.726, *p *=* *.011), and inclusion of data from the seasonal studies gave a similar relationship (Figure 3b; R = −.899, *p *= <.001). Thus, at sampling sites with relatively high prey diversity, the salmon and bullhead segregated in resource use and food resource partitioning was high, whereas when prey diversity was low, food resource partitioning was also low. Our sensitivity analyses did not alter the results, and the best model was also the model including only prey diversity as explanatory variable (AIC_c_ = 80.1; Table S4). Additionally, the model remains the same using bootstrapped data, corroborating a significant negative correlation between dietary overlap and prey diversity (R = −.991, *p *<* *.001).

**Table 3:** Summary of the best linear mixed‐effects model explaining variation of food resource partitioning between Atlantic salmon parr and alpine bullhead. Standard error = *SE*

	Value	*SE*	*t* value	*p* value
Intercept	95.02	18.64	5.096	<.001
Prey diversity	−75.21	23.75	−3.166	.011

Variables other than prey diversity could influence food resource partitioning (see Table 2), and model simulations after forward variable selection (see Table S3) suggest that the abundance of surface prey in the diet of salmon and fish abundance (salmon, bullhead, and Arctic charr abundance) may have had some influence on food resource partitioning. The abundance of surface prey in the diet of salmon gave a significant negative correlation with dietary overlap between the salmon and bullhead (Figure 3c; R = −.671, *p *=* *.024).

## 4 Discussion

There was a negative correlation between prey diversity and dietary overlap of salmon and bullhead, supporting the hypothesis that high prey diversity may enhance food resource partitioning between sympatric species and thereby facilitate their coexistence. In previous studies, high dietary overlap was observed between salmon and bullhead at a homogeneous river site with a low diversity of zoobenthos (Gabler & Amundsen, 1999), whereas strong dietary segregation was observed between salmon and European bullhead (*Cottus gobio* Linnaeus, 1758) in a more heterogeneous river that had relatively high diversity of zoobenthos (Gabler, Amundsen, & Herfindal, 2001). Thus, resource partitioning between salmon and bullhead species may be related to between‐river differences in prey diversity and habitat characteristics (Gabler & Amundsen, 1999; Gabler et al., 2001). The present study reveals that food resource partitioning between salmon and bullhead can vary within a river system, between sites at relatively short distances from each other, and between seasons at a given site, with resource partitioning being strongly dependent on prey diversity at different sites. Studies on other species have given indication that high prey diversity may enhance food resource partitioning (see, e.g., Hillebrand & Shurin, 2005; Jiang et al., 2008; Martin & Garnett, 2013; Zapata et al., 2005) and that competition for food is high when prey diversity is low (Barili et al., 2011; Hillebrand & Shurin, 2005; Targett, 1981). Our study corroborates these findings and supports the notion that high prey diversity may promote consumer coexistence through food resource partitioning.

**Figure 4:**
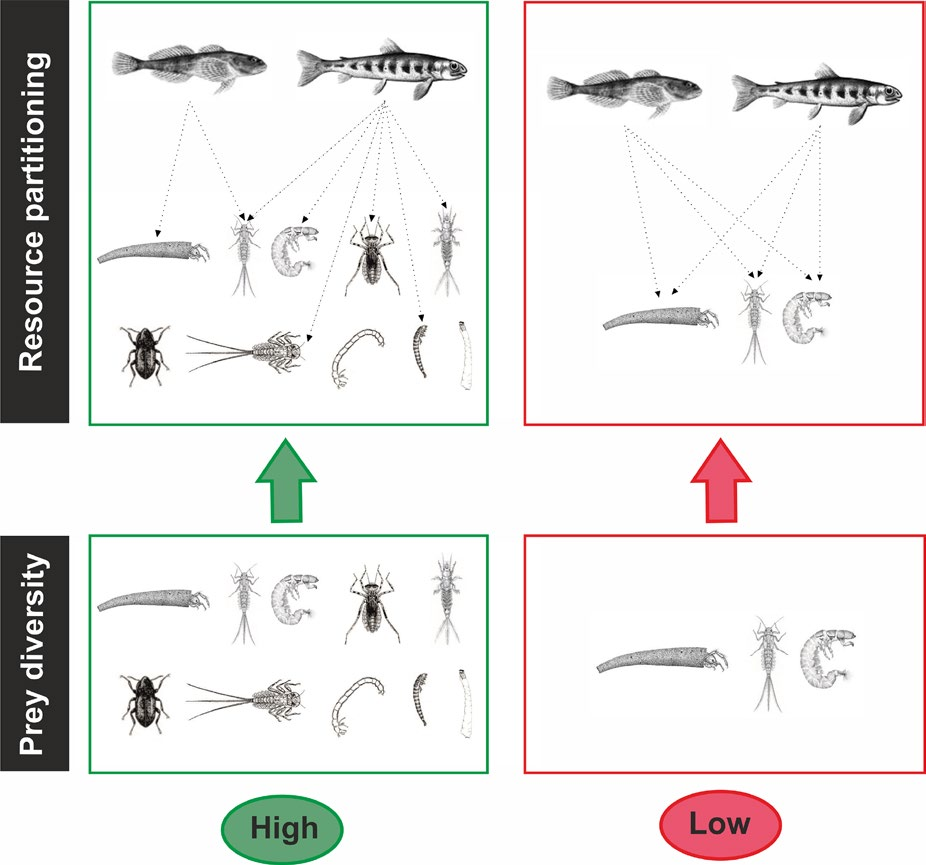
Schematic illustration of the potential influence of prey diversity on resource partitioning between two stream‐dwelling fish species in sympatry (here Atlantic salmon parr and alpine bullhead). For example, if prey diversity is low, it is probable that there will be strong competition because prey diversity is insufficient to allow sympatric consumers to specialize and segregate in prey use

It is hypothesized that prey diversity as it relates to competition for food resources and partitioning could have an influence on segregation and species coexistence in consumers with similar trophic niche requirements. Theoretical considerations that address relationships between dietary overlap, competition, and coexistence posit that competition forces sympatric species to diverge and segregate in resource use (Schoener, 1974, 1989), the weaker species may be excluded (e.g., Eloranta, Knudsen, & Amundsen, 2013; Nakano et al., 1999; Schoener, 1989), or ecologically similar sympatric species may converge and overlap in resource use (e.g., Cucherousset, Aymes, Santoul, & Céréghino, 2007; Keddy, 2001; Paterson et al., 2014; Wiens, 1993). These are seemingly contradictory standpoints. The first consideration encapsulates the competitive exclusion principle (Gause, 1934; Hardin, 1960) that has been widely accepted by many in the scientific community, and the second, although being more controversial, has also received some support (see, e.g., Bengtsson, 1991; Grant, 1972; terHorst, Miller, & Powell, 2010). We suggest that the apparent contradictions can be resolved if prey diversity is taken into account. Our reasoning is as follows: Competition for food may result in either high and low dietary overlap between sympatric consumers depending on prey diversity. If prey diversity is high, the competing species may segregate in resource use by specialization, for example, exploitation of *Glossosoma intermedium* (Klapalek 1892) by bullhead and use of surface prey and *Apatania stigmatella* (Zetterstedt 1840) by salmon. Under these circumstances, competition results in resource segregation through a low degree of dietary overlap, as predicted by classic niche theory (e.g., Jiang et al., 2008; Martin & Garnett, 2013; Schoener, 1989; Targett, 1981). The strength of competition may be low because the competing species have the possibility to minimize negative effects by segregating their use of food resources via specialization. On the other hand, if prey diversity is low, it is probable that there will be strong competition because prey diversity is insufficient to allow sympatric consumers to specialize and segregate in prey use (Figure 4). This complies with a situation that leads to a theoretical prediction that competition will result in high niche overlap if the species are symmetrical in their competitive abilities (Ågren & Fagerstrøm, 1984; Gilbert, 2012; Keddy, 2001), or if competition is very strong (Martin & Genner, 2009; Schoener, 1989; Wiens, 1993). Thus, the seemingly contrasting considerations about how competition affects dietary overlap may not be truly contradictory, but both may be valid depending upon the scale of prey diversity.

In addition to prey diversity, prey abundance might be a factor that influences the strength of competition (see, e.g., Triplet, Stillman, & Goss‐Custard, 1999). The intuitive expectation is that competition for food should be higher when resources are scarce than when they are abundant. Although prey abundance was a significant predictor variable in the forward stepwise procedure, other explanatory variables were more influential (see Table 2). In this regard, fish abundance could play a role in governing food resource partitioning. This is in agreement with previous works, demonstrating that fish abundance can exacerbate competition for food in fish assemblages (Elliott, 1994; Engelhard et al., 2013). It is important to note that the correlation between dietary overlap and fish abundance was not significant, so fish abundance may operate synergistically with prey diversity to enhance food resource partitioning (see models including fish abundance in Table S3). For example, Barili et al. (2011) reported that high fish abundance and diversity can promote trophic specialization in sympatric species, thereby enhancing food resource partitioning, and density‐dependent foraging behavior may occur when resources are limited (Sánchez‐Hernández & Cobo, 2013). Noteworthy, our interferences regarding the influence of density dependence on the competition for food should be taken with some caution because our analyses included the overall fish density regardless of fish size. Nevertheless, it is reasonable to posit that fish abundance may impact on the mechanisms involved in food resource partitioning as discussed earlier.

Our study revealed that surface prey were strongly represented in the diet of salmon at sites where dietary overlap between salmon and bullhead was lowest, suggesting that surface feeding may be a contributing factor that drives food resource partitioning between stream‐dwelling fish species (see, e.g., Dineen et al., 2007; Sánchez‐Hernández, Gabler, & Amundsen, 2016; Sánchez‐Hernández, Servia, Vieira‐Lanero, & Cobo, 2013). Although salmon and bullhead usually feed primarily on benthic invertebrates (Amundsen & Gabler, 2008; Gabler & Amundsen, 1999, 2010), our study clearly demonstrates that bullhead feed less on surface prey than do salmon, with the proportion of surface‐drift foragers being substantially higher in salmon than in bullhead (Sánchez‐Hernández et al., 2016). Thus, this study corroborates the flexibility of salmon adopting its foraging modes in relation to bullhead. The inference is that bullhead has a preference for foraging close to the bottom, whereas salmon may forage throughout the water column and can adopt different foraging modes to overcome competition with the co‐occurring species, but the lack of drift sampling did not allow us to assess whether or not this feeding behavior adopted by salmon is motivated by drift availability or food competition with bullhead. However, this flexibility is likely to be influenced by prey availabilities (e.g., Nakano et al., 1999; Sánchez‐Hernández & Cobo, 2013), leading to relationships that are influenced by benthic invertebrate diversity and the availability of surface prey. Diel patterns of feeding and habitat utilization have the potential to influence food resource partitioning between sympatric species (e.g., Crow et al., 2010; Kronfeld‐Schor & Dayan, 2003; Sánchez‐Hernández et al., 2011). However, previous studies in River Reisa revealed no strong segregation in foraging time (diel feeding rhythms) and space (habitat) between salmon and bullhead (Amundsen & Gabler, 2008; Gabler & Amundsen, 1999, 2010), which supports our main conclusion that prey diversity is the main driver of resource partitioning in these two species. Still, the capacity to forage at the water surface (surface feeding) by salmon needs to be acknowledged as a spatial segregation in feeding contributing to the observed resource partitioning between the two model species.

Prey diversity emerged as the strongest predictor of resource partitioning between salmon and bullhead, although resource partitioning was also influenced to some extent by surface prey use, and fish and prey abundances. Prey diversity and surface prey may have operated synergistically to enhance food resource partitioning between salmon and bullhead. Additional work will be needed to explore and enhance our understanding of how the interface between aquatic and terrestrial ecosystems influences ecological processes, such as resource partitioning.

## Conflict of interest

None declared.
