# Supplementary figures and images for: Prey diversity as a driver of resource partitioning between river‐dwelling fish species
Source: Ecol Evol. 2017 Feb 26;7(7):2058–68. doi: 10.1002/ece3.2793 (PMC5383502; doi:10.1002/ece3.2793)

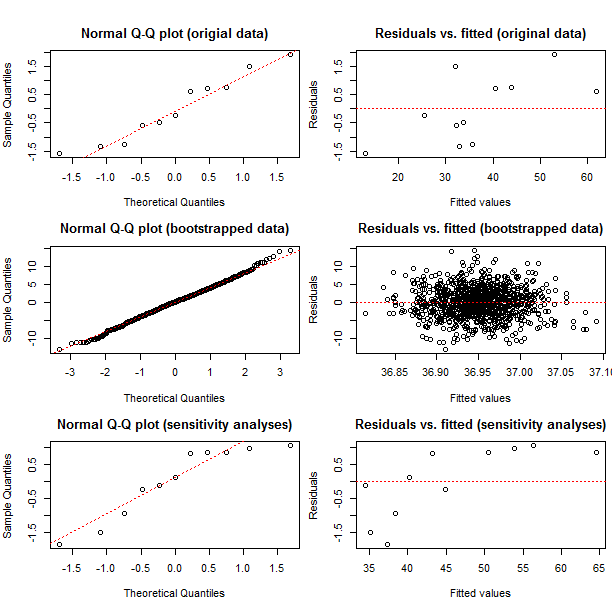

Supplement: Supplementary file 1 [file ECE3-7-2058-s001.tiff]
