# Supplementary material for: Prey diversity as a driver of resource partitioning between river‐dwelling fish species
Source: Ecol Evol. 2017 Feb 26;7(7):2058–68. doi: 10.1002/ece3.2793 (PMC5383502; doi:10.1002/ece3.2793)
Supplement: Supplementary file 2 [file ECE3-7-2058-s002.docx]

**Table S1.** Composition of potential benthic prey taxa in the environment. Data are presented as relative proportion of each prey taxon (%) for each sampling site.

|  | SS1 | SS2 | SS3 | SS4 | SS5 | SS6 | SS7 | SS8 | SS9 | SS10 | SS11 |
| --- | --- | --- | --- | --- | --- | --- | --- | --- | --- | --- | --- |
| Plecoptera |  |  |  |  |  |  |  |  |  |  |  |
| *Diura nanseni* | 0.8 | 0.9 | 0.4 | 0.5 | 0.7 | 3.6 | 0.7 | 4.9 | 0.7 | 1.8 | 4.6 |
| *Isogenus* sp. | – | 0.1 | – | – | – | – | – | – | – | – | – |
| *Taeniopteryx nebulaosa* | 0.3 | 0.4 | – | – | 4.2 | – | – | 0.1 | 0.2 | 0.4 | – |
| *Capnia* sp. | 13.1 | 15.4 | 0.5 | 8.2 | 2.1 | 16.5 | 3.1 | 54.6 | 4.7 | 32.8 | 43.9 |
| *Leuctra* sp. | 2.3 | 1.2 | – | 0.7 | 2.1 | – | 0.5 | – | – | – | 0.2 |
| *Leuctra digitata* | – | – | – | – | – | 0.2 | – | – | – | – | – |
| *Capnia*/*leuctra* sp. | – | 0.1 | – | – | – | – | – | – | – | – | – |
| *Nemoura* sp. | 0.1 | – | – | – | – | – | – | – | – | – | – |
| Plecoptera gen. sp. | 0.04 | – | – | – | – | – | – | – | – | – | – |
| Ephemeroptera |  |  |  |  |  |  |  |  |  |  |  |
| *Ameletus inopinatus* | – | 0.7 | – | 2.1 | – | 2.3 | – | 4.9 | 2.8 | 1.4 | 3.4 |
| *Baetis muticus* | 0.1 | 1.2 | 1.6 | 0.7 | 0.7 | 0.9 | – | 2.0 | – | 1.1 | 4.2 |
| *Baetis* spp. | 8.7 | 24.9 | – | 0.2 | 0.7 | 18.5 | 4.1 | 2.5 | – | 1.5 | 7.6 |
| *Heptagenia dalecarlica* | 2.7 | 2.9 | 0.7 | – | – | 5.7 | 2.1 | 3.0 | 1.4 | 4.1 | 6.8 |
| *Ephemerella aurivillii* | 14.6 | 17.0 | 16.2 | 3.7 | 7.0 | 9.8 | 5.9 | 6.6 | 41.3 | 7.5 | 5.5 |
| *E. mucronata* | – | – | – | – | – | – | – | – | – | 0.1 | – |
| *Heptagenia joerensis* | – | 0.7 | – | – | – | – | – | – | – | – | – |
| *Metretopus alter* | – | – | – | – | – | – | – | – | 0.2 | – | – |
| *Siphlonurus* sp. | – | – | 19.1 | – | – | – | – | – | – | – | – |
| Trichoptera |  |  |  |  |  |  |  |  |  |  |  |
| *Rhyacophila nubila* | 0.04 | 0.1 | 1.4 | – | – | 0.2 | 0.9 | – | 0.2 | – | – |
| *Glossosoma intermedium* | – | 0.6 | 1.3 | – | – | 0.4 | 0.2 | 0.6 | 0.2 | 0.6 | 1.3 |
| *Arctopsyche ladogensis* | 0.8 | 6.0 | 1.8 | – | – | 0.5 | 0.7 | 0.7 | 0.2 | 1.6 | 3.2 |
| *Polycentropus flavomaculatus* | 0.7 | 0.1 | 0.4 | – | – | 0.4 | 0.1 | 0.1 | – | 0.2 | 2.3 |
| *Apatania stigmatella* | 0.1 | – | 0.5 | 0.7 | – | 1.6 | 1.4 | 3.0 | 8.9 | 3.5 | 5.1 |
| Trichoptera sp. 1 | 0.1 | – | – | 0.2 | – | – | – | – | – | – | – |
| Trichoptera sp. 2 | – | – | – | – | – | – | 0.5 | – | – | 0.2 | – |
| Trichoptera sp. 3 | – | – | – | – | – | 0.2 | 0.1 | 0.4 | – | – | – |
| Trichoptera sp. 4 | – | – | – | – | – | 0.2 | – | 0.1 | – | – | – |
| Trichoptera sp. 5 | – | – | 0.2 | – | – | – | – | – | – | 0.3 | – |
| Mollusca |  |  |  |  |  |  |  |  |  |  |  |
| Sphaeriidae gen. sp. | 0.8 | 0.1 | – | 1.2 | – | 1.2 | 0.4 | 0.2 | – | 0.4 | 0.2 |
| *Lymnaea* sp. | 0.5 | – | 0.7 | – | 1.4 | 5.2 | 1.7 | 0.2 | 0.7 | 0.7 | – |
| Diptera |  |  |  |  |  |  |  |  |  |  |  |
| Chironomidae (larvae) | 50.5 | 20.5 | 45.2 | 73.2 | 58.0 | 26.2 | 66.0 | 13.4 | 27.0 | 38.0 | 8.7 |
| Chironomidae (pupae) | 0.8 | 0.7 | 0.4 | 0.7 | 1.4 | 0.5 | 4.8 | – | 1.2 | – | – |
| *Tipula* sp. | 0.2 | 0.1 | – | – | – | 0.5 | 0.5 | 0.1 | – | – | – |
| Ceratopogonidae gen. sp. | 0.3 | – | 0.4 | 3.5 | 1.4 | 0.2 | – | 0.2 | 0.2 | – | 0.2 |
| Diptera sp. 1 | 0.4 | 0.1 | 1.3 | – | – | 1.8 | 1.1 | 0.8 | 0.7 | 0.1 | 0.8 |
| Diptera sp. 2 | – | 2.0 | – | – | – | – | – | 0.2 | – | – | – |
| Coleoptera |  |  |  |  |  |  |  |  |  |  |  |
| *Elmis* sp. | 0.5 | 0.3 | 1.1 | 0.5 | 2.1 | 0.2 | 0.9 | – | – | 0.2 | – |
| Dytiscidae (adult) | – | – | – | 0.2 | – | – | 0.1 | – | – | – | – |
| Dytiscidae (larvae) | – | – | – | – | 0.7 | – | – | – | – | 0.5 | – |
| Coleoptera (larvae) | 0.04 | – | – | – | – | – | 0.2 | – | – | 0.1 | – |
| Other taxa |  |  |  |  |  |  |  |  |  |  |  |
| Oligochaeta gen. sp. | 0.3 | – | – | – | – | – | – | 0.1 | 0.7 | 0.3 | 0.4 |
| Hydracarina spp. | 1.2 | 2.2 | 2.0 | 3.7 | 7.0 | 3.4 | 3.6 | 0.8 | 4.7 | 2.0 | 1.1 |
| *Cottus poecilopus* | 0.04 | – | – | – | – | – | – | – | – | – | – |
| Collembola gen. sp. | – | – | – | – | – | – | – | – | 4.0 | – | 0.2 |
| Copepoda gen. sp. | – | – | – | – | 10.5 | – | – | – | – | 0.2 | – |
| Unidentified larvae sp. 1 | 0.1 | 1.2 | 4.5 | – | – | – | 0.1 | – | – | 0.1 | 0.2 |
| Unidentified larvae sp. 2 | 0.04 | 0.3 | – | – | – | – | 0.1 | – | – | – | – |
| Unidentified pupae | – | – | 0.4 | – | – | – | 0.1 | – | – | – | – |
