# Supplementary material for: Prey diversity as a driver of resource partitioning between river‐dwelling fish species
Source: Ecol Evol. 2017 Feb 26;7(7):2058–68. doi: 10.1002/ece3.2793 (PMC5383502; doi:10.1002/ece3.2793)
Supplement: Supplementary file 3 [file ECE3-7-2058-s003.docx]

**Table S2.** Diet abundance (%) of Atlantic salmon parr (Sal) and alpine bullhead (Bul). Data are displayed for sampling site (SS).

|  | SS1 | |  | SS2 | |  | SS3 | |  | SS4 | |  | SS5 | |  | SS6 | |  | SS7 | |  | SS8 | |  | SS9 | |  | SS10 | |  | SS11 | |
| --- | --- | --- | --- | --- | --- | --- | --- | --- | --- | --- | --- | --- | --- | --- | --- | --- | --- | --- | --- | --- | --- | --- | --- | --- | --- | --- | --- | --- | --- | --- | --- | --- |
|  | Sal | Bul |  | Sal | Bul |  | Sal | Bul |  | Sal | Bul |  | Sal | Bul |  | Sal | Bul |  | Sal | Bul |  | Sal | Bul |  | Sal | Bul |  | Sal | Bul |  | Sal | Bul |
| Plecoptera |  |  |  |  |  |  |  |  |  |  |  |  |  |  |  |  |  |  |  |  |  |  |  |  |  |  |  |  |  |  |  |  |
| *Diura nanseni* | 4.3 | 4.1 |  | – | 0.7 |  | 0.6 | – |  | 3.5 | 3.7 |  | 13.5 | 3.1 |  | 20.0 | 2.3 |  | 3.1 | 6.3 |  | 8.4 | 20.8 |  | 17.5 | 5.6 |  | – | 2.1 |  |  | 8.8 |
| *Capnia*/*leuctra* | – | – |  | – | 5.4 |  | – | – |  | 5.0 | 2.6 |  | 2.5 | 1.0 |  | 0.7 | 1.9 |  | 0.3 | – |  | 4.3 | 1.4 |  | – | 1.4 |  | – | 1.7 |  | – | – |
| *Leuctra digitata* | 4.3 | 8.3 |  | 2.5 | – |  | 2.2 | – |  | – | 0.6 |  | – | – |  | – | – |  | 0.7 | – |  | – | – |  | – | 1.2 |  | – | – |  | – | – |
| *Taeniopteryx nebulaosa* | 0.7 | – |  | – | – |  | 0.6 | – |  | – | – |  | – | – |  | – | – |  | – | – |  | – | 0.5 |  | – | – |  | – | – |  | – | – |
| Plecoptera sp. 1 | – | – |  | – | – |  | – | – |  | – | – |  | – | – |  | – | – |  | – | – |  | – | – |  | 1.6 | 0.7 |  | – | – |  | – | – |
| Unidentified (larvae) | – | – |  | – | – |  | – | – |  | – | – |  | – | – |  | – | – |  | – | – |  | – | 1.8 |  | – | – |  | – | – |  | – | – |
| Ephemeroptera |  |  |  |  |  |  |  |  |  |  |  |  |  |  |  |  |  |  |  |  |  |  |  |  |  |  |  |  |  |  |  |  |
| *Ephemerella aurivillii* | 12.1 | 7.8 |  | 2.5 | 13.0 |  | 21.7 | 7.4 |  | 6.0 | 0.8 |  | 6.2 | 5.0 |  |  | 3.3 |  | 14.6 | 45.7 |  | 6.2 | 2.1 |  | 3.2 | 6.5 |  | – | – |  | – | 1.3 |
| *Ameletus inopinatus* | – | – |  |  |  |  |  |  |  |  |  |  |  |  |  | 11.1 | 5.0 |  | – | – |  | – | – |  | – | – |  | – | – |  | – | 2.5 |
| *Heptagenia dalecarlica* | 20.0 | 5.1 |  |  | 1.3 |  | 7.8 |  |  | 15.0 | 6.9 |  | 10.0 | 2.4 |  | 10.8 | 0.1 |  | 5.8 | 10.8 |  | 10.1 | 3.0 |  |  | 2.4 |  | – | 15.5 |  | – | 10.0 |
| *Baetis* spp. | 17.1 | 1.2 |  | 2.5 | 0.1 |  | 2.0 | 4.0 |  | 2.3 | 3.2 |  | 1.5 | 3.7 |  |  | 12.3 |  | 1.4 | – |  | 1.4 | – |  | – | – |  | – | – |  | – | – |
| Unidentified (nympha) | – | – |  | – | – |  | – | – |  | – | – |  | – | – |  | – | 0.1 |  | – | – |  | – | 0.5 |  | – | – |  | – | – |  | – | – |
| Trichoptera |  |  |  |  |  |  |  |  |  |  |  |  |  |  |  |  |  |  |  |  |  |  |  |  |  |  |  |  |  |  |  |  |
| *Apatania stigmatella* | – | – |  | 1.3 | 0.1 |  | 3.9 | – |  | 10.0 | 0.9 |  | 6.8 | 2.1 |  | 17.3 | 1.8 |  | 44.5 | 5.9 |  | 35.5 | 2.7 |  | 56.1 | 3.5 |  | – | 18.5 |  | 32.8 | 9.8 |
| *Arcynopteryx compacta* | – | 0.8 |  | – | 0.2 |  | – | 15.4 |  | – | – |  | – | 2.5 |  | – | – |  | 0.2 | – |  | – | 13.0 |  | – | 13.0 |  | – | – |  | – | – |
| *Arctopsyche ladogensis* | – | – |  | – | 2.8 |  | – | – |  | 1.5 | 4.2 |  | – | 2.4 |  | – | 3.3 |  | 0.1 | – |  | – | – |  | – | – |  | 12.0 | 9.3 |  | – | 13.3 |
| *Glossosoma intermedium* | – | 1.8 |  | – | 7.6 |  | 4.4 | 18.4 |  | – | 11.8 |  | – | 39.0 |  | – | 6.4 |  | 0.5 | – |  | 17.8 | 31.9 |  | 10.0 | 30.4 |  |  | 12.4 |  | 5.6 | 29.2 |
| *Rhyacophila nubila* | 1.4 | 2.0 |  | – | – |  | 2.6 | 3.4 |  | 7.5 | – |  | – | – |  | – | – |  | 2.1 | – |  | – | – |  | – | – |  | – | – |  | 5.0 | – |
| *Polycentropus flavomaculatus* | – | – |  | – | 0.3 |  | – | – |  | – | 1.1 |  | – | – |  | – | – |  | – | – |  | – | – |  | – | – |  | – | – |  | – | – |
| Trichoptera sp. 1 | – | 2.9 |  | 10.0 | 1.8 |  | 8.3 | – |  | – | 2.4 |  | – | – |  | – | – |  | 1.2 | – |  | 3.2 | 1.8 |  | – | 0.6 |  | – | – |  | – | – |
| Trichoptera sp. 2 | 2.9 | 1.8 |  | – | 19.4 |  | – | – |  | – | – |  | – | 0.2 |  | – | – |  | 2.8 | 1.3 |  | – | – |  | – | – |  | – | 1.4 |  | 26.7 | 10.0 |
| Trichoptera sp. 3 | – | – |  | – | 1.5 |  | – | – |  | – | – |  | – | – |  | – | – |  | – | – |  | – | 1.7 |  | – | – |  | – | – |  | – | – |
| Trichoptera sp. 4 | – | 2.0 |  | – | 1.9 |  | – | – |  | – | – |  | – | – |  | – | – |  | – | – |  | – | – |  | – | – |  | – | – |  | – | – |
| Unidentified (larvae) | – | – |  | – | – |  | – | – |  | – | – |  | – | – |  | – | – |  | – | – |  | 2.1 | 4.6 |  | – | – |  | – | – |  | – | – |
| Unidentified (pupae) | – | – |  | – | – |  | 11.1 | – |  | – | – |  | – | – |  | – | – |  | 2.6 | – |  | 2.1 | – |  | – | – |  | – | – |  | – | – |
| Mollusca |  |  |  |  |  |  |  |  |  |  |  |  |  |  |  |  |  |  |  |  |  |  |  |  |  |  |  |  |  |  |  |  |
| *Lymnaea* sp. | – | – |  | – | 0.4 |  | – | – |  | – | – |  | – | – |  | – | – |  | 1.9 | 2.5 |  | 0.5 | – |  | – | – |  | – | 1.4 |  | – | – |
| Unidentified | – | – |  | – | – |  | – | – |  | – | – |  | – | – |  | – | – |  | – | – |  | 0.5 | – |  | – | – |  | – | – |  | – | – |
| Diptera |  |  |  |  |  |  |  |  |  |  |  |  |  |  |  |  |  |  |  |  |  |  |  |  |  |  |  |  |  |  |  |  |
| Chironomidae (larvae) | 28.6 | 56.1 |  | 3.8 | 34.9 |  | 2.8 | 3.4 |  | 35.3 | 45.6 |  | – | – |  | 10.7 | 19.4 |  | 0.7 | 8.6 |  | 5.3 | 6.8 |  | 5.6 | 4.3 |  | 68.0 | 32.0 |  | – | 1.0 |
| *Tipula* sp. | – | – |  | – | – |  | – | 29.0 |  | – | 4.2 |  | – | 0.8 |  | – | 25.1 |  | 1.3 | – |  | – | 4.0 |  | – | 13.6 |  | – | – |  | – | – |
| Sinulidae gen. sp. | 3.6 | 0.3 |  | – | 0.6 |  | 4.4 | 11.0 |  | – | – |  | – | 0.2 |  | – | – |  | – | – |  | – | – |  | – | – |  | – | – |  | – | – |
| Diptera sp. 1 | – | 0.5 |  | – | 2.9 |  | – | – |  | – | – |  | – | 2.7 |  | 18.3 | 12.9 |  | – | 5.6 |  | – | – |  | – | 7.1 |  | – | 5.5 |  | – | 14.3 |
| Unidentified (larvae) | – | – |  | – | – |  | – | – |  | – | – |  | 16.6 | 19.2 |  | – | – |  | 6.5 | 1.3 |  | – | 1.6 |  | – | – |  | – | – |  | – | – |
| Unidentified (pupae) | 3.6 | 0.3 |  | 1.3 | – |  | – | – |  | 1.0 | – |  | 0.5 | – |  | – | – |  | 0.6 | – |  | 1.1 | – |  | – | – |  | – | – |  | – | – |
| Coleoptera |  |  |  |  |  |  |  |  |  |  |  |  |  |  |  |  |  |  |  |  |  |  |  |  |  |  |  |  |  |  |  |  |
| *Elmis* sp. | – | 1.8 |  | 10.0 | 3.2 |  | 12.8 | 8.0 |  | – | – |  | – | – |  | 11.1 | – |  | 0.3 | – |  | 1.6 | – |  | – | 0.9 |  | – | – |  | – | – |
| Coleoptera (adult) | – | – |  | – | 0.4 |  | 2.2 | – |  | – | – |  | – | – |  | – | – |  | 4.5 | – |  | – | – |  | 6.0 | 5.8 |  | – | – |  | – | – |
| Unidentified | 1.4 | – |  | 1.3 | – |  | – | – |  | 7.0 | 6.4 |  | – | – |  | – | – |  | 0.2 | – |  | – | – |  | – | – |  | – | – |  | – | – |
| Other taxa |  |  |  |  |  |  |  |  |  |  |  |  |  |  |  |  |  |  |  |  |  |  |  |  |  |  |  |  |  |  |  |  |
| Chydoridae gen. sp. | – | – |  | – | – |  | – | – |  | – | – |  | – | – |  | – | 0.8 |  | – | – |  | – | – |  | – | – |  | – | – |  | – | – |
| Hydracarina spp. | – | – |  | – | – |  | 0.9 | – |  | – | – |  | – | – |  | – | – |  | – | – |  | – | – |  | – | – |  | – | – |  | – | – |
| Fish prey | – | 2.9 |  | – | 0.9 |  | – | – |  | – | – |  | – | – |  | – | – |  | – | 12.3 |  | – | 0.9 |  | – | 2.4 |  | – | – |  | – | – |
| Terrestrial insects | – | – |  | 65.0 | – |  | 11.7 | – |  | 6.0 | 2.4 |  | 42.5 | – |  | – | – |  | – | – |  | – | – |  | – | – |  | 20.0 | – |  | 30.0 | – |
| Aerial stages of aquatic insects | – | – |  | – | – |  | – | – |  | – | – |  | – | 9.0 |  | – | 1.7 |  | – | – |  | – | – |  | – | – |  | – | – |  | – | – |
| Spiders | – | – |  | – | – |  | – | – |  | – | – |  | – | – |  | – | – |  | – | – |  | – | – |  | – | 0.9 |  | – | 0.2 |  | – | – |
| *Culicoides* spp. | – | – |  | – | – |  | – | – |  | – | 2.2 |  | – | – |  | – | 0.1 |  | – | – |  | – | – |  | – | – |  | – | – |  | – | – |
| Unidentified sp. 1 | – | – |  | – | – |  | – | – |  | – | – |  | – | – |  | – | – |  | 2.1 | – |  | – | – |  | – | – |  | – | – |  | – | – |
| Unidentified sp. 3 | – | 0.1 |  | – | 0.5 |  | – | – |  | – | 1.1 |  | – | 0.9 |  | – | 3.5 |  | – | – |  | – | 0.8 |  | – | – |  | – | – |  | – | – |
| Stone | – | – |  | – | – |  | – | – |  | – | – |  | – | – |  | – | – |  | 2.2 | – |  | – | – |  | – | – |  | – | – |  | – | – |
| Twigs (vegetal) | – | – |  | – | – |  | – | – |  | – | – |  | – | 6.0 |  | – | – |  | – | – |  | – | – |  | – | – |  | – | – |  | – | – |
