# Supplementary material for: Prey diversity as a driver of resource partitioning between river‐dwelling fish species
Source: Ecol Evol. 2017 Feb 26;7(7):2058–68. doi: 10.1002/ece3.2793 (PMC5383502; doi:10.1002/ece3.2793)
Supplement: Supplementary file 4 [file ECE3-7-2058-s004.docx]

**Table S3.** Summary table for the ten best model simulations according to AIC_c_ values (the best model is the model with the lowest AIC_c_ values).

| Model | Intercept | Predictor variables | | | | | | | | AIC_c_ values |
| --- | --- | --- | --- | --- | --- | --- | --- | --- | --- | --- |
|  |  | Prey diversity | Niche breadth (alpine bullhead) | Arctic charr abundance | Surface prey (Atlantic salmon) | Size (Atlantic salmon) | Atlantic salmon abundance | Size (Alpine bullhead) | Alpine bullhead abundance |  |
| 1 | 95.02 | -75.21 | – | – | – | – | – | – | – | 82.4 |
| 2 | 96.04 | -69.59 | -1.02 | – | – | – | – | – | – | 85.4 |
| 3 | 94.85 | -76.16 | – | 0.68 | – | – | – | – | – | 86.8 |
| 4 | 86.62 | -58.09 | – | – | -0.30 | – | – | – | – | 87.2 |
| 5 | 112.20 | -70.49 | – | – | – | -0.36 | – | – | – | 87.2 |
| 6 | 103.80 | -82.0 | – | – | – | – | -0.68 | – | – | 87.7 |
| 7 | 126.10 | -79.59 | – | – | – | – | – | -0.45 | – | 88.1 |
| 8 | 92.83 | -76.97 | – | – | – | – | – | – | – | 90.0 |
| 9 | 66.52 | – | -5.63 | – | – | – | – | – | – | 91.0 |
| 10 | 100.80 | -65.60 | – | – | -0.40 | – | -1.31 | – | – | 91.1 |
