# Supplementary material for: Prey diversity as a driver of resource partitioning between river‐dwelling fish species
Source: Ecol Evol. 2017 Feb 26;7(7):2058–68. doi: 10.1002/ece3.2793 (PMC5383502; doi:10.1002/ece3.2793)
Supplement: Supplementary file 5 [file ECE3-7-2058-s005.docx]

**Table S4.** Summary of the best linear-mixed effects model for our sensitivity analyses (excluding surface prey from the dietary analyses from the linear-mixed effects modeling) explaining variation of food resource partitioning between Atlantic salmon parr and alpine bullhead. Standard error = SE.

|  | Value | SE | *t* value | *P* value |
| --- | --- | --- | --- | --- |
| Intercept | 82.89 | 16.33 | 5.07 | <0.001 |
| Prey diversity | -48.46 | 20.81 | -2.33 | 0.045 |
